# CORRELATES OF DENTAL CARE UTILIZATION AMONG US INFORMAL DEMENTIA CAREGIVERS

**DOI:** 10.1093/geroni/igad104.0051

**Published:** 2023-12-21

**Authors:** Weiyu Mao, Bei Wu, Wei Yang

**Affiliations:** University of Nevada, Reno, Reno, Nevada, United States; New York University, New York City, New York, United States; University of Nevada Reno, Reno, Nevada, United States

## Abstract

Informal dementia caregivers tend to report more negative health impacts from the caregiving role, higher levels of unmet needs, and lower levels of service utilization compared to caregivers of persons with other types of illnesses. There remains a gap in knowledge of understanding unique correlates of dental care utilization among informal dementia caregivers. As guided by the Andersen model, this study examined correlates of dental care utilization among U.S. informal dementia caregivers. Data came from the Behavioral Risk Factor Surveillance System collected in 2016, 2018, and 2020 (n=2,931). Respondents were selected when they reported providing regular care or assistance to a friend or family member who has Alzheimer’s disease, dementia, or other cognitive impairment disorder. Dental care utilization was dichotomized into “yes” versus “no” within the past year. Caregiving-related characteristics included caregiver role (e.g., spouse, adult child, other relative, and friend), intensity of care, duration of care, and type of care. Other correlates included caregiver characteristics, health behaviors, and physical and oral health needs. Findings from stepwise logistic regression models demonstrate that being a spousal caregiver, providing more than 20 hours of care per week, being male, having high school or less education, being low-income, unemployed, current smoker, having no health insurance, no current alcohol use, or less than adequate sleep was associated with a lower likelihood of vising a dentist within the past year. The findings suggest targeting spousal caregivers and those who provide intensive care to improve dental care-seeking behaviors in dementia caregiver support intervention strategies.

